# Author response to: Comment on: Cachexia index for prognostication in surgical patients with locally advanced oesophageal or gastric cancer: multicentre cohort study

**DOI:** 10.1093/bjs/znae135

**Published:** 2024-05-30

**Authors:** Leo R Brown, Andrew B Crumley, Richard J E Skipworth

**Affiliations:** Clinical Surgery, University of Edinburgh, Royal Infirmary of Edinburgh, Edinburgh, UK; Department of General Surgery, Forth Valley Royal Hospital, Larbert, UK; Department of General Surgery, Forth Valley Royal Hospital, Larbert, UK; Academic Unit of Surgery, University of Glasgow, Glasgow Royal Infirmary, Glasgow, UK; Clinical Surgery, University of Edinburgh, Royal Infirmary of Edinburgh, Edinburgh, UK

*Dear Editor*

We thank Li *et al*. for their interest in our study^1^ and welcome the opportunity to discuss the points raised.

Consideration of clinical over pathological staging was intentional in light of the study’s aims. We wished to assess the prognostic value of cachexia index (CXI) as an adjunct to tumour stage during pretreatment decision-making. Pathological staging is more reliable and prognostic than clinical staging, but would only be known after surgery and thus unable to influence treatment planning. This rationale was highlighted in our discussion with comment of this limitation to other models. It would be interesting, however, to investigate the relationship between CXI and downstaging during neoadjuvant therapy.

CXI’s association with other ‘host’ variables was less of a concern in the context of our study. The cohort proceeded on a curative pathway following subspecialist multidisciplinary team review. Thus, advanced age (median difference approximately 4 years) or major co-morbidities were insufficient to preclude planned treatments. Rather than robustly isolating the causal treatment effect of low CXI, our aim was to evaluate CXI as a composite marker of the host’s response to cancer, with other host markers inevitably contributing towards that response. The multidimensional nature of these co-variates, when applied through techniques such as propensity score matching, results in a greater risk of overfitting. The association with lower albumin level and higher neutrophil–lymphocyte ratio was not noteworthy, given that these markers form part of the calculation for CXI and would inevitably contribute to it being lowered.

Reported alcohol consumption would indeed have been interesting if available. Unlike smoking, estimated alcohol intake is infrequently recorded in patients’ notes unless consumption is notably high. However, most of our patients had oesophageal adenocarcinoma, which has a lesser association with alcohol consumption than oesophageal squamous cell carcinoma. Data on alcohol consumption are being collected as part of an ongoing prospective cachexia characterization study (REVOLUTION Surgery; NCT05642819) which should provide a platform to explore such questions.
